# Towards a Functional Understanding of PGO Waves

**DOI:** 10.3389/fnhum.2017.00089

**Published:** 2017-03-03

**Authors:** Jarrod A. Gott, David T. J. Liley, J. Allan Hobson

**Affiliations:** ^1^Centre for Human Psychopharmacology, Swinburne University of TechnologyMelbourne, VIC, Australia; ^2^Division of Sleep Medicine, Harvard Medical SchoolBoston, MA, USA

**Keywords:** magnetoencephalogram, rapid eye movements, pontine-geniculate-occipital waves, 5-HT, vision

## Abstract

Ponto-Geniculo-Occipital (PGO) waves are biphasic field potentials identified in a range of mammalian species that are ubiquitous with sleep, but can also be identified in waking perception and eye movement. Their role in REM sleep and visual perception more broadly may constitute a promising avenue for further research, however what was once an active field of study has recently fallen into stasis. With the reality that invasive recordings performed on animals cannot be replicated in humans; while animals themselves cannot convey experience to the extent required to elucidate how PGO waves factor into awareness and behavior, innovative solutions are required if significant research outcomes are to ever be realized. Advances in non-invasive imaging technologies and sophistication in imaging methods now offer substantial scope to renew the study of the electrophysiological substrates of waking and dreaming perception. Among these, Magnetoencephalogram (MEG) stands out through its capacity to measure deep brain activations with high temporal resolution. With the current trend in sleep and dream research to produce translational findings of psychopathological and medical significance, in addition to the clear links that PGO wave generation sites share, pharmacologically, with receptors involved in expression of mental illness; there is a strong case to support scientific research into PGO waves and develop a functional understanding of their broader role in human perception.

## Introduction to PGO Waves

Ponto-Geniculo-Occipital (PGO) waves are distinctive wave forms that are typically identified as propagating activity between three key brain regions, being the Pons, Lateral Geniculate Nucleus and Occipital Cortex. They have been confirmed and studied extensively in cats, rats and primates but never in humans, as the ethical implications for performing invasive recordings at three concurrent brain locations has not made such research justifiable or indeed practical. Rare occasions where neurosurgical medical procedures have been performed around these key regions have indeed opened the prospective window for simultaneous invasive recording for scientific purposes, however to date this has only confirmed the Pontine elements of the phenomenon and as such, it can only be conservatively said that humans exhibit *P waves* (Lim et al., 2007).

PGO waves were initially observed in 1957 in anesthetized cats (McGaugh, 2012), and have since been comprehensively studied in this species, to a lesser extent in rats (Kaufman and Morrison, 1981), and in non-human primates such as Macaques and Baboons (Cohen and Feldman, 1968; Vuillon-Cacciuttolo and Seri, 1978). However, the network of brain regions that PGO waves propagate through does vary from species to species, with only the *Pons* and to some extent the *Lateral Geniculate Body* of the Visual Thalamus forming a common neurophysiological location across all mammalian species tested thus far. The PGO wave profile strongly resembles that of a sub-threshold epileptiform burst (Elazar and Hobson, 1985), and like other epileptiform bursts they may propagate and spread throughout the brain, potentially via top-down activation by the Amygdala and the Prefrontal Cortex. Beyond this, little is known except that they are also early predictors for the onset of REM sleep, and are reproducible *in vivo* through Acetylcholine microinjections to the brainstem (Vanni-Mercier and Debilly, 1998; Márquez-Ruiz and Escudero, 2009). However there are numerous indications that PGO waves function beyond sleep physiology alone, and may have additionally important implications for the systems that underpin waking perception and healthy psychological function (Hobson, 2009; Hobson and Friston, 2012). While typically associated with REM sleep, PGO waves also correlate with saccadic eye movement in waking, albiet at lower electrophysiological amplitude, across all mammalian species tested (Nelson et al., 1983).

PGO waves have been observed to occur several seconds after heightened activity within the feline pontine subregion known as the *caudolateral peribrachial area (C-PBL;* Datta, 1997*)*, which receives inhibitory serotonergic projections from the *Raphe Nuclei*, which in turn is thought to partly mediate the specific pharmacological links between Serotonin (5-HT) and PGO wave genesis. As expected, pharmacological agents that deplete synaptic 5-HT such as *Fenclonine* and *Reserpine* have been shown to increase PGO wave amplitude to match that of REM sleep (Brooks et al., 1972; Jacobs et al., 1972), while similarly, 5-HT_1A_ agonists have been observed to suppress PGO wave activity altogether (Quattrochi et al., 1993). Interestingly, 5-HT_2A_ agonists have not been observed to affect PGO activity in either direction (Sanford et al., 1997), indicating a potentially confounding mechanism of action between visual phenomena produced through dreaming and those produced via classical psychedelics that rely on strong activations of this receptor subtype. This finding had initially complicated earlier models which had assumed psychoactive distortion of visual process would occur through modulation of a common receptor. However contemporary perspectives that allow macroscopic system behaviors to be considered without linear dependence on specific lower order variables now render this observation considerably less problematic to the broader role of PGO waves in human vision.

Given the anticipated role that 5-HT receptor subtypes play in the expression of psychopathological illness, such as body dysmorphic disorders and unipolar depression (Celada et al., 2004; Bailer et al., 2007), the role these receptors also play in REM sleep (Ansseau et al., 1993; Landolt and Wehrle, 2009), and the growing consensus that features of agency and self-reflection characterized in psychopathology depend on network level changes similarly produced REM sleep (Lysaker et al., 2011; Filevich et al., 2015), there is considerable clinical potential in further understanding the mechanisms that underpin PGO waves. Indeed, PGO wave research may eventually provide an important link in explaining how the systems that allow internally generated imagery to unconsciously modulate and override retinal visual input synchronize in healthy perception, and subsequently break down in psychopathology. Thus a functional understanding into the genesis of unconscious perceptive systems, and the role of PGO waves within these systems, is likely to be of relevance in understanding important dynamical aspects of conscious experience.

At present there is no holistically compelling model to justify how retinal sensory input is overridden, producing dreaming, creative experience and psychotic hallucination in human populations. What explanations do exist are largely confined to narrow disciplinary approaches, while these in turn remain relatively niche and often lack translational relevancy outside their specific fields. Thus a purely Psychodynamic account explains little more than a purely computational one—however much each coheres with its disciplinary techniques and traditions. In order to fully understand and comprehend the complex issues surrounding false inferences in human sensory perception, an interdisciplinary approach is required, and this must thoroughly investigate the phenomenon in question, using both sound theoretical understanding and sophisticated imaging methods. Since there is general optimism that human and feline anatomical structures responsible for PGO wave generation share many similarities (Vanni-Mercier and Debilly, 1998; Fernández-Mendoza et al., 2009), the translational value of feline studies conducted in previous decades remains a significant asset to future research. This is especially the case when attempting to develop a theoretical understanding of the relationship between PGO wave activity and macroscopic, observable behavior. However, human subjects are ultimately required if the gap between theoretical and functional is ever to be closed, and experiments must be designed to falsify assertions made within the allowance of contemporary ethical constraints. As such, a detailed investigation into human PGO waves using non-invasive technologies such as Magnetoencephalogram (MEG) appear to be the best prospect for achieving such progress.

## PGO Waves in Animals

### Cats

**Figure 1:**
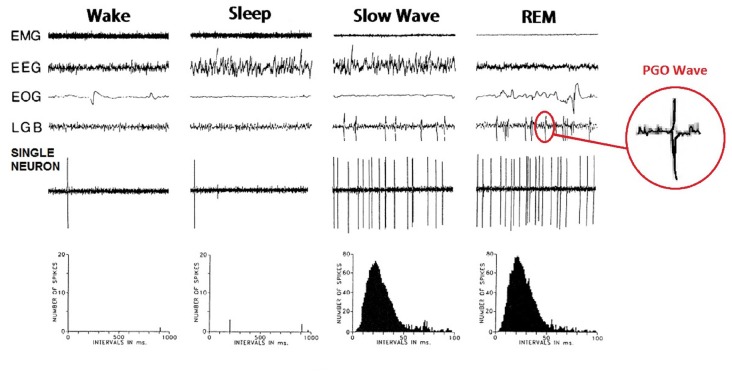
**Ponto-Geniculo-Occipital (PGO) waves in cats.** Measurements of single nonbursting type PGO on-state Neuron, located in the *caudolateral peribrachial area (C-PBL)* of a cat. Widespread distribution of PGO wave activity correlates with a triggering-neuron transitioning from tonic to high frequency activity. Image adapted from Datta and Hobson (1994).

PGO waves were first observed in the feline brain, during invasive electrophysiological investigations into sleep in that species. The observed phenomenon was subsequently named after the three regions they most prominently appeared, being the Pons, the Geniculate Nucleus and the Occipital Cortex (Jouvet and Michel, 1959; Mikiten et al., 1961; Brooks and Bizzi, 1963; Mouret et al., 1963). They can be observed as both single waves, occurring predominantly in NREM sleep and not time-locked to eye movement, and wave bursts, which occur primarily during REM and correlate strongly with saccadic movements (see Figure 1). Both display a biphasic shape and last for 60–120 ms with an amplitude of 200–300 μV. PGO wave bursts typically come in clusters of 3–5 and occur at approximately 30–60 spikes per minute both preceding and during REM sleep (Bowe-Anders et al., 1974; Datta, 1997). PGO Waves have also been recorded in a state of wakefulness, albeit at lower amplitude and frequency (Brooks, 1968; Stern et al., 1972).

The neurons involved in generating and propagating PGO waves can be divided into two groups, being the *Executive Neurons* that trigger PGO wave onset and propagate similar electrophysiological activity to other brain regions; and *Modulatory Neurons* that respond to fluctuations in neuromodulators (Datta, 1997). Neuromodulators are thought to facilitate some degree of involvement of forebrain and limbic structures in PGO wave generation and behavior, and while not directly being involved in their primary generation, have significant influence on how PGO waves are spread and maintained. Executive Neurons are primarily located within the *C-PBL* of the *Dorsolateral Pons* (Datta, 1997), while Modulatory Neurons are located across disparate brain regions, including the Prefrontal Cortex, Amygdala, Suprachiasmatic Nucleus, as well as vestibular and auditory cell groups. As expected; lesions to the *Peribrachial Area* eliminate primary PGO wave generation, while a wide range of aminergic partial and full agonists, cholinergic agonists and reuptake inhibitors similarly affect PGO wave generation in a variety of ways, from delaying to prolonging onset, increasing or decreasing their amplitude and potentially even influencing how PGO waves and brain networks interact.

PGO waves occur in both Lateral Geniculate Bodies, nearly simultaneously (Nelson et al., 1983). However the Geniculate Body in one hemisphere will typically produces a wave of considerably higher amplitude, a few milliseconds earlier than the other. These higher amplitude waves have been labeled “Primary” PGO waves, while the lower amplitude ones that follow them have been labeled “Secondary” waves (Laurent et al., 1974; Cespuglio et al., 1975). When PGO waves appear in relation to a REM saccade, wave generation reliably correlates with the direction of eye movement, with Primary waves occurring in the ipsilateral^1^ Geniculate body, and Secondary waves in the contralateral body to the saccade. Primary waves in turn result from activity in a group of *Executive Neurons* known as state-on “burst cells” (McCarley et al., 1978; Nelson et al., 1983), which precede Primary wave generation by approximately 25 ms, and also have their lateral activation correlate strongly with saccade direction (Nelson et al., 1983; Datta, 1997).

### Rodents

In rodents, PGO waves are generally referred to as pontine-waves or *P waves*, and do not appear to propagate in the same manner as in species with more developed visual systems. Nonetheless, the term PGO wave is still used, in broader reference to the interspecies phenomenon. In this species, waves have been observed to last for 70–180 ms with amplitude of 70–280 μV (Kaufman and Morrison, 1981) and were initially recorded in both the *Locus Coeruleus* and *Tractus Mesencephali* of the Pons (Marks et al., 1980) and the Cerebellum (Farber et al., 1980). Rats appear to produce PGO waves following direct electrical stimulation of the Amygdala (Deboer et al., 1998) and this is thought to indicate a relationship between dreaming and specific epochs of alertness. PGO waves have been shown to function somewhat independently of the acoustic startle response, and may in this sense indicate endogenous activation of the retina independent of a behavioral response (Kaufman, 1983).

Localized injections of Carbachol to the rat brainstem have been successfully used to isolate PGO wave generation to the dorsal *Nucleus Subcoeruleus* of the Pons (Datta et al., 1998) which suggests an important link between PGO generation and brain areas such as the Amygdala, Hippocampus, Entorhinal Cortex and Piriform Cortex, which all receive dense projections from this location. This finding highlights a potentially important relationship between PGO wave generation and other cognitive functions, including sensorimotor function and memory consolidation. Further investigations into rats have revealed important links between the PGO waves and memory-related gene expression in both the Amygdala and Hippocampus (Datta et al., 2008), while another study has correlated PGO wave density with successful consolidation fear extinction following traumatic stress (Datta and O’Malley, 2013).

## PGO Waves and Neuropharmacology

The influence of neuropharmacological compounds on PGO wave generation and behavior is complex and only partially understood, with the development of considerably more sophisticated theoretical models required to account for the often confounding experimental data. Existing models have combined observational findings with mathematical modeling, often involving *Predictive Coding* (Hobson, 2014). Since there exists an experimental gulf between the quality of subjective experience produced from human subjects and the ethical constraints of administering psychomimetic compounds to non-animal cohorts; carefully designed research is required to translate the findings and theoretical implications of feline and rodent studies into a human context. The difficulties in producing translational research from feline to humans is further compounded by the logistical barriers in accessing pharmacological compounds that may affect PGO waves in humans, as many are designated as controlled substances and are not legally accessible.

Conservatively speaking, substances that globally deplete 5-HT in the feline brain are considerably likely to promote high amplitude, REM-like PGO waves, even while awake. This can be effectively achieved through acute administration of vesicular monoamine transporter (VMAT) blockers such as *Reserpine* (Brooks and Gershon, 1972) in addition to chronic administration of the tryptophan hydroxylase inhibitor *Fenclonine* (Ruch-Monachon et al., 1976). Administration of 5-HT_1A_ agonists DPAT^2^ and mCPP^3^ appear to have an indirect inhibitory effect on PGO wave generation, potentially by modulating the influence of the startle mechanism during the initiation of REM sleep (Sanford et al., 1994), however these effects do not occur independent of animal behavior, and as such, do not reveal much about PGO wave modulation since causation cannot be established. Likewise, 5-HT2_A_ agonists and antagonists have failed to directly modulate PGO wave activity in any significant way (Sanford et al., 1994), a finding that was not anticipated and difficult to reconcile with expectations and predictions produced using existing models.

One example where the direct relationship between 5-HT receptor subtypes, internally generated perception and electrophysiological phenomenon has proven notably difficult to understand involves the well-known 5-HT2_A_ receptor agonist and recreational psychedelic, *lysergic acid diethylamide* (LSD). Many studies have shown LSD to suppress PGO wave generation in felines (Brooks, 1975; Ruch-Monachon et al., 1976), even in the presence of potent PGO wave promoters such as *Reserpine* (Züger et al., 1978). However, the hypothesis currently remains open regarding the precise mechanisms by which these substances induce their hallucinatory visual effects, with some anticipating a more complex mechanism involving NMDA antagonism (Aghajanian and Marek, 2000), while others anticipate a dopaminergic mechanism instead (Marona-Lewicka et al., 2005; Seeman et al., 2005). As such, the role that 5-HT plays in promoting certain visual phenomenon in one context while suppressing it in another creates many problems. It may be the case that the relationship between internally generated imagery, PGO waves and psychoactive 5-HT receptor agonists is considerably more complex than once thought, with further research and more innovative models needed to establish context for empirical findings that now span multiple academic disciplines. While PGO waves may ultimately facilitate information generated within the *Default Mode Network* to augment and override the information produced from the retina, there may be other mechanism by which this can occur, and indeed PGO waves may not present a mandatory precondition for this to happen. Untangling the complex interactions between these two approaches (electrophysiological and psychopharmacological) may ultimately require human participants with an ability to subjectively report their experience.

## PGO Waves in Humans

In humans, PGO waves have been hypothesized to hold significance across important and diverse domains of cognition, such as learning (Datta, 2006), brain maturation and network organization (Amzica and Steriade, 1996). Given the demonstrated importance REM sleep shares with these functions, this observation is not surprising, and thus the true importance and scientific relevance of human PGO waves is at best, only partially known. Despite the potential value of further exploration and investigation into this area, experiments on human subjects have not matched those conducted on felines. This can broadly be seen as a twofold problem: the complexity and relative novelty of suitable non-invasive technology renders such research somewhat niche and unattractive, while logistical complexity and risk of performing invasive recordings on humans significantly limits the scope of the experimental paradigm, and very little can be safely confirmed other than the existence of the waves themselves. Despite these difficulties, some progress has been made on both fronts, and as such; PGO wave research may soon undergo a significant resurgence.

An invasive study into human PGO waves was performed, during a clinical neurosurgical operation to treat Parkinson’s Disease symptoms, through the stereotactic implantation of a quadripolar DBS electrode into the *pedunculopontine nucleus* of the *Pontine tegmentum* of a single patient (Lim et al., 2007). The patient was subsequently monitored over a 24 h period, 2 days post operatively, during which normal REM sleep stages were observed and recorded. Results indicated that biphasic waves of 150–200 ms duration and 10–15 μV amplitude, bearing an “unmistakable morphologic resemblance” to the P-Waves of rats. However, potentially owing to the pathological condition of the patient, or indeed remnants of the anesthetic procedure itself, these P-Waves were not consistently associated with REM Sleep, occurring during only 41% of REM and only seldom during waking eye saccades. Despite this, the concluding remarks of the study were that the *Activation Synthesis Hypothesis* (see “Current Theory” Section) that underpins this interpretation of PGO wave function was largely supported; and that mass non-invasive recordings of healthy subjects remained the most viable method of translating feline models into human ones.

**Figure 2:**
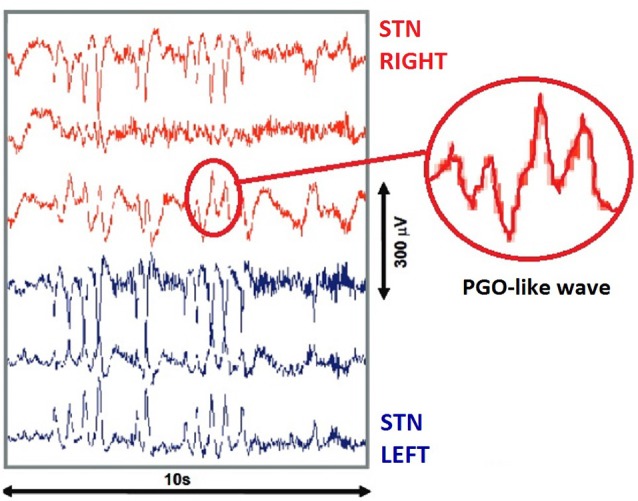
**Human invasive recordings.** PGO like waves recorded from the *Subthalamic Nucleus* of a surgical patient during REM sleep, using deep brain stimulation electrodes. Red lines (top) indicate three recording sites in the Right STN, while Blue lines (bottom) indicate three recording sites in the Left STN. Image adapted from Fernández-Mendoza et al. (2009).

Shortly after this study, another was conducted using a similar cohort of patients with Parkinson’s Disease, however in this case the sample size was considerably expanded to include 12 individuals (Fernández-Mendoza et al., 2009). Deep Brain Stimulation electrodes were surgically implanted under general anesthetic with recordings commencing 84 h after surgery. In this study, the target site was instead the *Subthalamic Nucleus*, and produced results considerably more in line with existing feline models. Both PGO wave *Singlets* and *Clusters* were evident, with durations of approximately 350 ms and amplitudes of 100 μV (see Figure 2). Clusters occurred in groups of approximately 3–13, with a density of 18–27 spikes per minute. Much like feline models, PGO wave clusters were observed to be closely related to REM Sleep and additionally demonstrated an observable fast oscillatory Subthalamic Beta activity in the 13–35 Hz range. Importantly, this study also demonstrated a degree of homogeneity between feline and human PGO wave function, in finding that PGO wave singlets precede REM Sleep onset by 30–90 s in both species.

A single-neuron study was most recently conducted, using depth-electrode EEG, in a cohort of 13 epilepsy patients (Andrillon et al., 2015). Potentials in the human Medial Temporal Lobe having a morphology similar to feline PGO waves were reliably observed time-locked to sleep REM, and added considerable support to the hypothesis that PGO waves propagate throughout the brain, and far beyond the three key regions from which they have traditionally been observed. While this study did not provide unequivocal evidence of the PGO wave phenomena in humans, it none the less gave support to the anticipated existence and purported complexity of these phenomena.

## Non-Invasive Evidence for PGO Waves in Humans

To date, there have been very few attempts to find non-invasive evidence for PGO waves in humans, however those that have proceeded have shown promise. The only non-invasive imaging technology that has both the temporal and spatial resolution to potentially isolate and measure deep brain activity with sufficient fidelity to produce the characteristic electrophysiological PGO wave shape, is at present the MEG. However, many other non-invasive technologies, such as fMRI and positron emission tomography (PET) are capable of playing important roles in providing indirect evidence to support the existence of this phenomenon, for instance through recording of general brain activity time-locked to REM. Such observations may contribute to understanding the nature and extent of the PGO wave generating network, their respective overlap with REM generation sites (Miyauchi et al., 2009) and the relationship with limbic regions such as the Amygdala (Miyauchi et al., 2009) and the Parahippocampal Gyrus (Peigneux et al., 2001; Miyauchi et al., 2009). Due to the widespread availability of such technologies, and their demonstrated capability for recording neuronal activation deep within the brain, the potential to explore the phenomenon of human PGO waves from a range of complementary non-invasive technologies appears promising.

Functional MRI in combination with polysomnographic recording has shown some promise in this regard. One study (Miyauchi et al., 2009) demonstrated activations in the Pontine Tegmentum, Ventroposterior Thalamus and V1 of the Occipital Cortex, directly preceding onset of REM, in 13 of the 17 subjects tested. Additional activity was observed in the Anterior Cingulate, Parahippocampal Gyrus and Amygdala during phasic REM stages. Self-paced saccades in total darkness produced no specific changes, indicating that the observed activity related to phenomenal dream content, thus supporting the hypothesis that PGO waves may comprise the origins of such content. This observation was supported by an earlier study (Wehrle et al., 2005) which utilized both fMRI and polysomnography but did not analyze event-related activity specifically time-locked to saccades. In spite of this, it produced similar results, specifically; activations in the Ventroposterior Thalamus and V2 of the Occipital Cortex in 8 out of 11 subjects.

A study conducted using PET likewise supported the existence of PGO wave like activity in humans (Peigneux et al., 2001) by combining recordings of hemodynamic change with polysomnographic activity, in 12 subjects. This study found notable activity during REM in the Ventroposterior Thalamus and V1 of the Occipital Cortex, with additional activity in a number of limbic regions and the Parahippocampal Gyrus. Importantly, like Miyauchi et al. (2009) this study showed no significant activity during waking self-directed saccades, supporting the hypothesis that PGO waves play an important role in the generation of visual content that REM saccades subsequently process. However like Wehrle et al. (2005) this study did not time-lock changes to specific polysomnographic events, as PET lacks the temporal resolution to do so in any case. Since these technologies invariably rely on changes in hemodynamic activity, as a proxy for neural activity, their capacity to comprehensively demonstrate the existence of purely electrophysiological phenomenon such as PGO waves will remain limited, but nevertheless valuable to future research.

**Figure 3:**
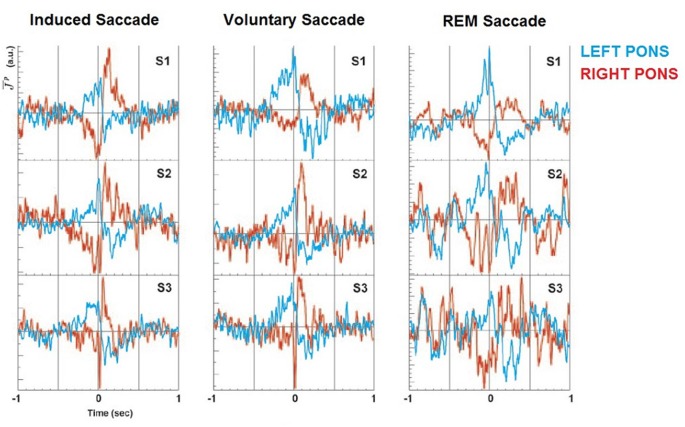
**PGO waves using Magnetoencephalogram (MEG).** Non-invasive recordings of the *mid-Pontine Nuclei* during leftward saccades in humans, over three conditions. PGO wave like activity clearly evident in hemisphere ipsilateral to saccade direction. S1–S3 indicate subjects, colors denotes laterality. Image reproduced from Ioannides et al. (2004).

MEG has both the potential spatial and temporal resolution to advance the research conducted using hemodynamic measurements, with the additional advantage that it directly records electrophysiological activity. This has enormous potential, when combined with structural MRI and simultaneous EEG and EOG to analyze event-related electrophysiological changes. However due to the complexity of the imaging forming process and data analysis, and—until recently—the severe computational overheads required to process MEG data, very little has been done to fully capitalize on the MEG to scientifically investigate this phenomenon. One study conducted in 2004 found preliminary evidence for PGO like activity in the Human Pons (Ioannides et al., 2004), during three kinds of saccade, including REM and self-directed waking (see Figure 3). Importantly, it indicated high amplitude spiking activity in the same lateral hemisphere as the direction of the saccade, indicating a degree of homogeneity with feline models produced using invasive recordings (Hobson and Friston, 2012). However the study was limited in that it only included three participants. Source-space images were produced using Magnetic Field Tomography (MFT)^4^ and subsequently overlayed on an fMRI topographical map constructed using Statistical Parametric Mapping to produce a spatially accurate representation of Pontine activity during saccades (Ribary et al., 1991). This study was able to record electrophysiological phenomenon that bore a distinct resemblance to the characteristic PGO wave profile, something only possible using invasive depth electrode placement to date.

The techniques used in this study could be replicated or adapted to future studies, with a variety of methods available to produce deep source localization of brain activity using electromagnetic measurement, including contemporary beamforming techniques (Litvak et al., 2010; Quraan et al., 2011). The challenges when using electromagnetic correlates to infer activity at depth within the brain, however, appear to center around the creation and detection of so-called “ghost” sources which may call the validity of such measurement into question (Attal and Schwartz, 2013). This term refers to electromagnetic sources of activity in inactive, or inconsequentially active brain regions, that become unintentionally observed through the very techniques necessary to isolate subcortical activity functionally coupled to cortical activity. This, however can be in large part ameliorated, using an increase in trial numbers, or in this case by improving the experimental paradigm through greater use of modeling. As such it may be necessary to effectively interpret measured activity within the context of predictable and incontrovertible physiological measurements such as EOG, within the context of existing physiological models relating to sleep.

## Current Theory

The significance of PGO waves was first appreciated by observing the behavior of surgically lesioned cats. A lesion to the brainstem, eliminating the *muscle atonia* associated with sleep, thus resulted in cats in a REM state rising from supine posture and pouncing on imaginary prey, hiding or orienting towards nonexistent threats (Morrison, 2014). This led to the understanding that REM Sleep and the phenomenal experience of dreaming potentially served the biological purpose of entraining waking experience, by simulating threatening or difficult environments so that learning might be achieved without physical exposure to risk (Revonsuo, 2000). Subsequent investigations into the relationship between REM and memory consolidation appear to have supported this idea (Datta, 2000; Smith and Rose, 2000). The development of this theory shifted popular conceptions of dreaming consciousness away from the popular Freudian one, where dreams were considered encoded messages from the subconscious (Freud, 1931), and similarly away from a behaviorist one, where according to orthodox interpretations, dreams do not exist at all (Malone et al., 2003). The culminating theoretical consensus produced from this research focus still remains open, however, and the central idea continues to evolve to accommodate relevant findings from many other fields, and as technological advances progressively allow this hypothesis to be tested.

Current theory borrows from the *Default Mode*^5^ concept (Greicius et al., 2003), and proposes that higher-order regions and functional networks within the brain are able to synthesize internal simulations of external sensory information, and that these simulations in turn may be integral to the emergence of higher-order cognitive function (Hobson et al., 2014). The biological process of REM Sleep, as such is thought to involve a partial disintegration of these processes—resulting on the one hand in the inability to critically reflect on one’s surroundings, having lost the very capacity to synthetically form the representations necessary to analyze them; whilst simultaneously experiencing a domain of visually simulated reality that is completely alien to and outside personal agency or control. This has necessitated the conceptualization of consciousness as existing in a *Primary* and *Secondary*^6^ sense (Hobson and Voss, 2011); with the functional changes occurring during REM being akin to something of a reversal of the phylogenic and ontogenic process by which these two domains had first come to be seamless. This would entertain the idea that the joining of purely experiential and synthetic components of the mind, undoubtedly produced through millennia of evolution, become structurally undone on a nightly basis in order that both can function more effectively in unison when waking life returns.

**Figure 4:**
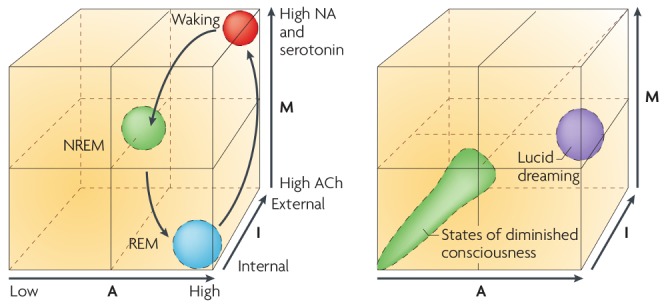
**The AIM Model.** A multidimensional states pace model that illustrates how “phenomenal experience” can arise as a dynamic process, from oscillatory activity across multiple subcomponents of brain function. A: Activity of brain, or given brain region. I: Source of sensory information (internal/external). M: Neuromodulation. Image reproduced from Hobson (2009).

The AIM (see Figure 4) model was one attempt to illustrate that dreaming and waking consciousness not only exist on the same spectrum of brain activity; but that an endless combination of brain state changes are indeed possible—both within and without healthy parameters. This suggests that unhealthy biological function, such as organic psychosis and various other psychopathologies, could be conceptualized as unintended changes to the biological processes that underpin the transitions between wakefulness and sleep (Hobson and Voss, 2010). This was further expanded to provide a biological basis for *Lucid Dreaming*^7^, which although once doubted, now has a strong theoretical and empirical basis (Voss et al., 2009). The AIM model was in part an evolution of the *Activation Synthesis Hypothesis* (Hobson and McCarley, 1977) which proposed that dreams were the result of spontaneous brainstem activation within sleep states, and that these experiences constituted the brain’s own attempt to interpret meaningless information. Later interpretations of this theory, particularly those relating to the AIM model, have added flexibility to accommodate the existence of agency within dream genesis, and thus allow for the existence of dream meaning, by reconceptualizing such control as a form of *Active Inference* emerging through discrete top down processes.

**Figure 5:**
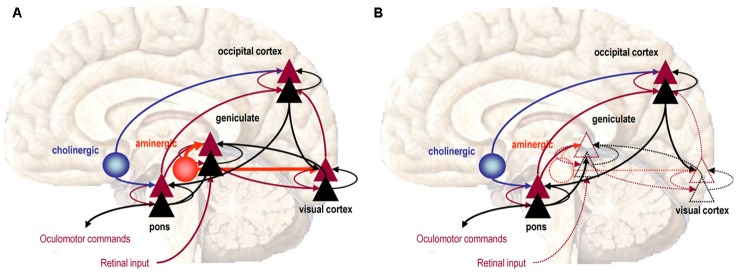
**Predictive coding and active inference. (A)** Waking perception is modeled as a series of forward connections between the *Lateral Geniculate Body* and the *Visual Cortex*, facilitated by the presence of Serotonin (5-HT), thus allowing retinal input to ascend. **(B)** The absence of 5-HT in sleep dampens the postsynaptic sensitivity of superficial pyramidal neuron groups, progressively reducing the capacity for retinal information to ascend through the *Visual Cortex* and become processed into vision, which now forms though feedback connections. Image reproduced from Hobson and Friston (2012).

Theoretical neurobiological concepts, such as *Predictive Coding*^8^ (Rao and Ballard, 1999), have subsequently been employed to justify the potential for *Active Inference* within the context of dreaming perception (Hobson and Friston, 2012). This has allowed theoretical demonstration (see Figure 5), by way of computational simulation, to justify how two fundamentally distinct aspects of conscious perception can overlap and dynamically interact to produce seamless experience and agency in the case of waking, and the disintegration of these qualities in psychosis and REM Sleep. *Active Inference* further implies that the poverty of retinal information, in conjunction with the necessity for the brain to produce accurate and believable sensory experience in the smallest available time, has necessitated a system of perception where prior expectations and memory, encoded within the neural synapse itself, actively and pre-emptively participate in bringing vision into awareness. The precise center of stability where the top-down control of sensory predictions is refrained from over-processing retinal input, but likewise, ensures that it is not under processed, is thought to go to the very heart of the brain’s attempt to maintain functional psychopathological health (Hobson et al., 2014).

The role of PGO waves is hypothesized to be integral to how information is exchanged and recurrently passed between dynamically gated neurophysiological regions and networks, to facilitate waking and dreaming perception as an evolutionarily instantiated maintenance process (Hobson, 2009). One interpretation holds that each PGO wave exists as a discrete epoch in which visual information is experienced, and essentially instructs the eye to orient towards perceived content during the earliest stages of unconscious perception. Another interpretation holds that each PGO wave acts as a *corollary discharge* from the retinal saccade, and merely indicates to the rest of the brain that the eyes have moved and visual information can be soon expected. Without further experimentation on human subjects, differentiating between these interpretations will remain philosophically and experimentally problematic, and as such, will likely impede the translation of this research into scientific and clinical relevancy. While this broad and disparate body of investigation has arguably started with PGO waves; in order that it may advance it must certainly return to them, and decisively attempt to understand the processes they subserve within a human behavioral context.

## Future Directions

Despite recent advances in computational and physiological models of the Visual Cortex, visual perception in humans is still poorly understood, with no clear consensus about how neuronal processes result in the production of vision as it is phenomenally experienced (Gordon, 2004). Since many of the current, conflicting interpretations run into near-unsolvable philosophical questions, such as free will and the causality of human agency on human behavior, none of these models have escaped the scrutiny they have attracted (Firestone and Scholl, 2015), and the question of causality in visual perception remains as ever open to interpretation. The evolving body of theory that underpins this research focus has largely attempted to steer clear of these philosophical questions by accepting aspects of both top-down and bottom-up paradigms of human visual synthesis as reasonable in their own right, and has attempted to find compromise in the notion that aspects of each may ultimately be responsible for the way human vision is experienced. Since the fundamental issue in producing a cohesive theory regarding the role of top-down synthesis of visual sensation inevitably returns back to the problem of scientific falsifiability of subjective claims, there are grounds to sidestep this challenge by focusing on domains of human experience where visual synthesis is so clearly present that such existence can be established *a priori*, without controversy.

Examples of these would include dreaming, pharmacologically induced hallucination and volitional recruitment of imagination, as these are all relatively difficult to quantify using empirical measures and yet are reasonably open to subjective reporting within the scientific standards of research. This effectively reverses the onus of proof on the weakest aspects of this particular body of theory, being the complex and arduous task of establishing that sensory experience actually takes place in a given individual, and instead proceeds to explore and investigate these phenomenon from the perspective from where they can safely be assumed to exist. As such, objective data can reasonably be assumed to represent these states under the correct conditions. Future investigations will seek to produce falsifiable evidence for perhaps the single least controversial argument in favor for top down synthetic perception, being the phenomenal existence of a human imagination, and subsequently use these findings to develop a more nuanced and challenging series of questions to further guide the research paradigm on the nature of human sensory experience.

Future theoretical modeling could relate to the existence of a synthetic imagination, experienced inexclusively through dreaming, which would be correlated with physiological brain mechanisms that effectively facilitate the brain’s ability to override its own retinal input. PGO waves are expected to play a central role in this process. This interpretation is one where the human brain produces visual experience through a balance of inputs originating both externally via the retina, and internally, via the physiological structures from which PGO waves are simultaneously produced. The precise compromise that actually translates into human experience, may in part be determined by the functional behavior of PGO waves within the visual system. Future investigations into the role of PGO waves in psychosis and psychopathology, could be served through a research focus on pharmacological hallucination, as generally only in such cases can the generated percept be mistaken for real, and result in the kind of hazardous fear response that classically affords psychopathology its stigma. In such cases, it is expected that the phenomenon of veridicity that accompanies an internally generated percept may represent only the first in a series of changes, that also include changes to attention, working memory and theory of mind, which collectively enable the phenomenon of synthetic inference to evolve into the macroscopic definition of psychopathology.

In light of these considerations, future directions in PGO wave research will necessitate the simultaneous development of a theoretical model for how the brain achieves both certainty and stability in its own visual content, and is likewise prevented from becoming pulled into runaway reactions to its own synthetic percepts. While the various neurological systems involved in generating dreams appear to simultaneously promote and contain this shift towards a volatile experience of mind; psychoses and pharmacological hallucination owe their precise danger to the very fact they exist outside this self-contained system and thus can fully recruit human action into the servitude of an otherwise synthetic reality. As such, there is a strong potential for this work to eventually be of translational relevance to mental health and psychopathological research, as both the cognitive systems and cognitive changes that underpin each could turn out to be remarkably similar. These theoretical investigations may provide fertile grounds for future advancements in both the scientific understanding of perception, and the subsequent treatment of psychopathology. Before these can be realized, however, the phenomenon of human PGO waves must be thoroughly studied and understood within a reconceptualized theoretical model. With the current ethical and technical constraints involved in existing invasive approaches to identify this phenomenon in humans, the future of PGO wave research undoubtedly lies in broad ranging studies that combine behavioral, pharmacological and cognitive experimentation on human subjects, using non-invasive methods such as fMRI and MEG.

## Author Contributions

All authors were involved in the conception, review of relevant literature, writing, editing and final approval of this work.

## Conflict of Interest Statement

The authors declare that the research was conducted in the absence of any commercial or financial relationships that could be construed as a potential conflict of interest.

## Footnotes

^1^Ipsilateral: Meaning *same side*. A saccade from left to right would produce a Primary wave in the Right LGB, and a Secondary wave in the Left LGB, and vice versa.

^2^8-hydroxy-2-(n-dipropylamino)tetralin: Relatively specific full 5-HT_1A_ and 5-HT_7_ agonist.

^3^meta-Chlorophenylpiperazine: Nonspecific 5-HT_1A_, 5-HT_1B_ and 5-HT_2_ agonist.

^4^Magnetic Field Tomography (MFT): A non-linear solution for solving the inverse problem in biomagnetic brain recordings. Can produce localized current densities in 3D space, from which neural activity in specific Regions of Interest (ROI) can be inferred (Ioannides et al., 1995).

^5^Default Mode: A conceptual interpretation of brain function that describes the apparent capability of the brain to generate its own content, in the absence of applied attention to the external world. This capacity is thought to originate from brain regions that have recently evolved in humans, that in turn may constitute a biological basis for advanced processes such as cognitive empathy and volitional use of memory. The term was coined following the accidental observation of spontaneous and unexpected blood flow changes in various brain regions during functional MRI, between stages where sensory information was scheduled for presentation. The observed network of activity was initially thought to comprise the "default" mode of activity of the brain, when not being used. However the term now exists largely in denotation of a parallel system of brain activity that operates independently of feed-forward sensory networks.

^6^Primary and Secondary Consciousness: An interpretation that overlaps somewhat with the concept of the *Default Mode Network*, but relates more specifically to the phenomenon of dreaming, and the dynamic relationship between lucid and reflexive aspects of self-agency. Primary Consciousness describes consciousness as it is experienced during dreaming, and presumably by humans during infancy, and by many animals: a stream of sensory experience, where choices are made but not necessarily subjectively experienced, and where reflection on one’s own existence and thought process (or that of others) is notably absent. Secondary Consciousness in turn relates to directed, volitional aspects of mental experience, which are largely synthetic and self-reflective in nature, and allow the phenomenal experience of choice through the generation of context, and through the exploration of hypothetical propositions relating to Primary Consciousness itself (Hobson and Voss, 2011).

^7^Lucid Dreaming: An experience of dreaming where self-agency and self-reflection are experientially returned to the individual, ostensibly resulting in the phenomenon of full consciousness within an artificial world. In many cases this regained control is accompanied by full or partial control of the dreaming world, and may include the sudden capability to fly, or to change aspects of the dream world through force of will. This has historically been a controversial subject, presumably due to its rarity, and its long association with religions and mystical practice dating back through human history. In contemporary scientific understanding, the onset of lucidity is associated with activation of a key region within the *Default Mode Network*, and the subsequent expansion of *Secondary Consciousness* within the dream state. Dream Lucidity is producible through training and auto-suggestion, or via transcranial induction of Gamma activity within these frontal regions (Voss et al., 2014).

^8^Predictive Coding: A model of perception and cognitive function originally described in Helmholtz’s *unconscious inference* and later given empirical grounding through concepts applied from *machine learning*. The premise of Predictive Coding in a cognitive sense is that the brain operates in a hierarchy, with unconscious processes such as formation of vision eventually giving rise to conscious ones, such as attention. In this view, higher layers of this hierarchy exist in a constant state of prediction, regarding the information that will feed forward from unconsciously processed perception. In turn, these higher layers exert a degree of control over behavior, and over the unconscious systems perception itself, by returning back down the hierarchy a quality of contextual meaning that allows unconscious perceptions to attain a degree of certainty and coherence. Where higher states in the hierarchy predict falsely, they receive prediction errors and over time, up or down regulate the certainty of their predictions. In terms of visual perception: hallucinations and delusions may result from either excessive, or alternatively insufficient predictive certainty within the system.
